# Updating Edwin Chadwick's seminal work on geographical inequalities by occupation

**DOI:** 10.1016/j.socscimed.2017.11.055

**Published:** 2018-01

**Authors:** Mark A. Green, Danny Dorling, Richard Mitchell

**Affiliations:** a Department of Geography & Planning, University of Liverpool, Liverpool, L69 7ZT, UK; b School of Geography and the Environment, University of Oxford, Oxford, OX1 2DL, UK; c Institute of Health and Wellbeing, University of Glasgow, Glasgow, G2 3QB, UK

**Keywords:** Edwin Chadwick, Occupation, Geography, Inequality, Mortality, Premature

## Abstract

To honour the 175^th^ anniversary of Edwin Chadwick's seminal ‘Report on the Sanitary Conditions of the Labouring Poor’, we update Chadwick's famous analysis of geographical differences in occupational based inequalities. Much of the field of Health Geography owes both its direction of development and its initial impetus to his 1842 report. The report presented evidence for the importance of local context to health, with individuals of the lowest occupations in Rutland living longer than individuals of the highest occupations in Liverpool. Here we update the 1842 analysis using data from the Office of National Statistics on individual mortality records by occupation (2010-12) and population data from the 2011 Census. Sex-specific directly standardised premature (16-74) mortality rates were calculated for hierarchical occupational categories similar to Chadwick's categories, for the nearest equivalent areas to those used in Chadwick's report. Although there is no longer consistent evidence on individuals in the lowest occupational group having lower mortality rates than those in the highest group, there were clear social gradients in mortality within each area and the extent of these inequalities varied between areas. Individuals who live in Rutland had lower premature mortality rates across each occupational group compared to the other areas. Our results demonstrate that while life expectancy has nearly doubled since Chadwick's report, social and spatial inequalities in health have persisted. We suggest that Chadwick's legacy on the importance of locality continues.

## Highlights

• We update Edwin Chadwick's seminal work to honour its 175 year anniversary.
• Social and spatial inequalities in health have remained consistent over 175 years.
• Premature mortality rates were highest in the lowest occupational group.
• Occupational based inequalities still varied spatially, as they also did in 1842.
• Our results show that Chadwick's legacy on the importance of locality continues.

## 1 Introduction

175 years ago, Edwin Chadwick published his seminal work ‘Report on the Sanitary Conditions of the Labouring Population of Great Britain’ (Chadwick, 1842). His painstaking documentation of statistical evidence of social and spatial inequalities in health and insanitary conditions was one of the earliest examples providing evidence based public health advice, and the findings provided the foundation for later advances made in the field by key figures such as William Farr. The Chadwick Report's findings, that poor sanitation conditions were associated with poor health, were controversial at the time and saw the Poor Law Committee (which commissioned the report) disown Chadwick. However, the report laid the groundwork for the introduction of the 1848 Public Health Act, a key piece of legislation which saw improvements in sanitation to tackle the causes of multiple infectious diseases (particularly cholera) and resulted in large improvements in population health (Szreter, 1997, Hamlin and Sheard, 1998, Krieger and Birn, 1998).

One key piece of evidence from the Chadwick Report was a single table which highlighted the importance of geography in identifying and understanding health inequalities. Chadwick utilised data on the average age of death by occupational group for five areas within England (Table 1) (Hanley, 2002). He did this to make a persuasive argument about the importance of local context in affecting health outcomes. The data showed both differences in ‘life expectancy’ by occupation (i.e. individuals further down the social gradient lived shorter lives), and that these patterns varied geographically. There was a clear interaction between geography and socioeconomic position; individuals of the lowest occupational group in Rutland could expect to live longer lives compared to those of the highest occupational group in Liverpool.

**Table 1:** Average age of death for occupation group by location (after Chadwick, 1842).

Location	Professional Trades	Tradesmen	Labourers
Rutland	52	41	38
Leeds	44	27	19
Liverpool	35	22	15
Manchester	38	20	17
Bolton	34	23	18

For the first time, it became clear that geographical context was important to consider alongside an individual's socioeconomic position (Hanley, 2002). During Chadwick's era, geographic context mattered partly because the cities he studied were, at the extreme, ‘cesspits’ rife with outbreaks of infectious diseases due to insanitary conditions (e.g. Cholera flourished due to a lack of clean water sources or the safe disposal of human waste killing thousands at a time), high level of pollutants due to unregulated industry, and overcrowded slum housing facilitating the spread of diseases. In contrast, Rutland was an ‘idyllic’ rural settlement set aside from the problems and squalor of Victorian cities. Much of the field of Health Geography today owes its direction of development and initial impetus to this single piece of evidence. It moved debates beyond simply describing geographical inequalities, towards identifying and explaining the numerous ways in which geographic context influences health outcomes.

On the 175th anniversary of the report, we update this important piece of evidence by examining the extent to which geographical inequalities still vary by occupational-based mortality rates, and whether the importance of local context seen in Chadwick's era still persists today.

## 2 Materials and methods

It is a legal requirement that all deaths in England are registered. Information on all deaths are compiled into a database by the Office for National Statistics (ONS) (Devis and Rooney, 1999). We were granted access to an anonymised version of the database which included data on individual deaths capturing age, sex, cause of death, occupation and residence (postcode). We extracted deaths for the calendar period 2010-12.

Occupation was captured as the last profession an individual held and was recorded using the National Statistics Socio-economic Classification (NS-SeC). Since all individuals who were aged between 16 and 74 had complete coverage in the database, we restricted the focus of our study to these ages only (i.e. premature mortality in adults) unlike Chadwick who considered all ages. We used the three group version of NS-SeC since these groups best approximated Chadwick's categories (as well as having a clear hierarchy allowing ordered relative comparisons). The categories were: ‘Higher’ (e.g. managerial and professional professions), ‘Intermediate’ (e.g. clerical, sales and small employers), and ‘Lower’ (e.g. routine and semi-routine occupations). A limitation of the occupational data is that the deceased's last profession was reported by the individual who registered the death. This may have introduced a misclassification error if insufficient detail was provided or the profession failed to reflect an individual's true socioeconomic position (e.g. they had changed occupation late in life) (Alderson, 1972). One strength of our use of broad occupational groups was to minimise the potential risk of misclassification bias as far as possible.

Sex-specific population counts by NS-SeC group were collected from the 2011 Census (we used these data as proxies for 2010 and 2012 as well). Sex-specific directly standardised mortality rates (per 100,000 population) were then calculated for each occupational group for each Local Authority to account for differences in the age composition between places (including 95% Confidence Intervals). We calculated the standard population for England using ONS, 2014 Census population statistics during the age-standardisation process.

We calculated the Spearman's Rank Correlation Coefficient between the average age of death in Chadwick's data (Table 1) and our estimates of premature mortality rates for males (for equivalent occupation groups individually) to examine how similar they are between the two periods. Since few females worked in 1842, it was appropriate to only test the association for males. We used Spearman's Rank Correlations rather than Pearson Coefficient as the data do not follow a linear association (Harris, 2016).

All analyses were completed using R and analytical scripts can be provided upon request.

## 3 Results

**Fig. 1:**
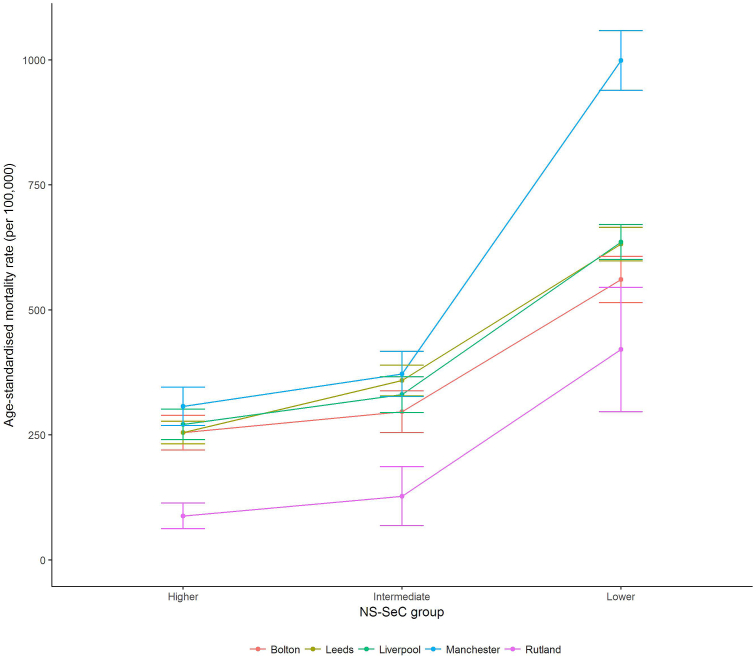
Male directly standardised premature mortality rates per 100,000 population (including 95% CIs) by occupation group for Chadwick's locations (2010-12).

**Fig. 2:**
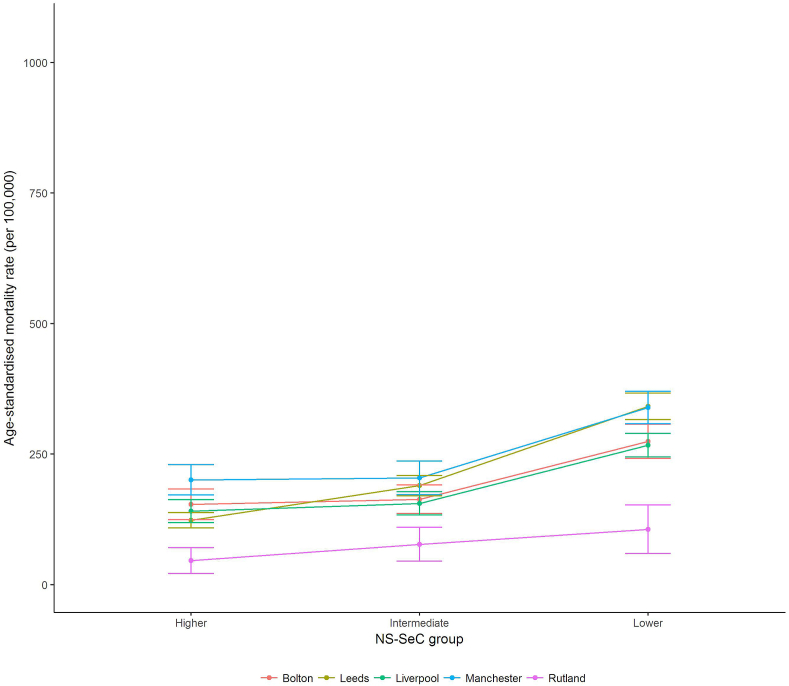
Female directly standardised premature mortality rates per 100,000 population (including 95% CIs) by occupation group for Chadwick's locations (2010-12).

Table 2 presents sex-specific directly standardised premature mortality rates by location for each occupational group (we have also plotted these results in Fig. 1, Fig. 2 to aid interpretation). There is evidence of a clear social gradient by occupation, whereby premature mortality rates were larger in the ‘lower’ occupational group compared to the ‘higher’ occupational group. These mortality differences by social group were consistent within location and sex, with premature mortality rates being roughly 2-3 times larger in the ‘lower’ occupational group compared to the ‘higher’ group.

**Table 2:** Directly standardised premature (16-74) mortality rates per 100,000 population (including 95% CIs) by occupation group for Chadwick's locations (2010-12).

Location	Males			Females		
	Higher	Intermediate	Lower	Higher	Intermediate	Lower
Rutland	88.0 (62.6–113.5)	127.6 (68.6–186.5)	420.7 (296.4–545.1)	46.1 (21.0–71.1)	77.3 (45.0–109.6)	106.0 (59.5–152.4)
Leeds	254.5 (232.0–277.0)	358.7 (327.8–389.7)	631.5 (597.8–665.3)	123.2 (108.4–138.0)	189.7 (170.1–209.2)	341.4 (316.1–366.7)
Liverpool	271.0 (240.5–301.5)	330.6 (294.6–366.5)	635.7 (600.8–670.7)	141.0 (119.0–162.9)	155.5 (133.3–177.7)	267.0 (244.5–289.5)
Manchester	307.0 (268.5–345.6)	372.0 (327.0–417.0)	998.5 (938.7–1058.2)	200.6 (171.6–229.6)	204.6 (172.5–236.7)	339.0 (308.0–370.0)
Bolton	254.6 (219.9–289.3)	296.5 (254.9–338.1)	560.8 (514.6–607.0)	153.8 (124.5–183.1)	163.5 (136.2–190.9)	274.3 (241.6–307.1)
England	213.6 (211.5–215.7)	309.9 (306.7–313.1)	447.0 (444.3–450.3)	132.8 (131.2–134.6)	156.4 (154.3–158.4)	220.9 (218.7–223.1)

Premature mortality rates also varied considerably *between* the geographical locations. Since fewer people now die under the age of 75 compared with 1842, due to improvements in the standard of living and medical progress, the confidence intervals for some of our estimates shown in Table 2 are wide. However, they are not too wide to prevent conclusions being drawn.

Rutland had the lowest premature mortality rate compared to the other locations, and this result was consistent within occupational group (i.e. the confidence intervals for the equivalent occupational group specific premature mortality rates in other locations did not overlap with Rutland). The magnitudes of these geographical differences were substantial, with premature mortality rates 2-3 times higher than compared to the other locations within occupational-group (other than for males of ‘lower’ occupational group where the magnitude of the effect size was smaller). There were smaller differences in premature mortality rates between the other locations. While Manchester had consistently higher premature mortality rates (particularly for the ‘lower’ occupational group), the confidence intervals typically overlapped with those for other locations.

Examining the differences between the ‘higher’ and ‘lower’ occupational groups by location in a similar vein to Chadwick's study revealed important insights. For males, the premature mortality rate for the ‘lower’ occupational group in Rutland was larger than both the premature mortality rates for the ‘higher’ and ‘intermediate’ occupational groups in the other locations. It suggests that the geographical inequalities have narrowed compared to Chadwick's era, since individuals in the lowest occupational groups in Rutland no longer have better health outcomes than those in the highest occupational groups. The premature mortality rate for the ‘intermediate’ group in Rutland was, however, smaller than the premature mortality rate for the ‘higher’ groups in all other locations (the confidence intervals do not overlap). It suggests that there still exists some form of interaction between location and socioeconomic position, similar to what Chadwick observed in 1842.

For females, the premature mortality rate for the ‘lower’ occupational group in Rutland was lower than the premature mortality rate for the ‘higher’ group in each of the other locations. Although the result would follow Chadwick's findings, the confidence intervals for Rutland overlapped when compared to each location other than Manchester, suggesting that the result was not statistically significant. When comparing the premature mortality rate for the ‘intermediate’ group in Rutland to the premature mortality rates for the ‘higher’ groups in the other locations, the premature mortality rate in Rutland was typically lower, with the confidence intervals overlapping in Leeds only. These results also present evidence to support Chadwick's assertion on the importance of locality, and also follow those observed for males.

Premature mortality rates were consistently lower for females compared to males within each location and occupational group. The difference in premature mortality rates between the ‘higher’ and ‘lower’ occupational groups was consistently wider, both in absolute and relative terms, for males compared to females. These results suggest that occupational-based inequalities are more pronounced for males, and that this association is consistently geographical.

Calculating the Spearman's Rank correlation between the values for men within Table 1, Table 2 for the equivalent occupational group produced coefficients of; -0.5 for the ‘higher’ group, -0.7 for the ‘intermediate’ group and -0.8 for the ‘lower’ group. These values suggest that the direction of health inequalities by geographical area and social class have remained broadly similar between two periods 175 years apart.

## 4 Conclusions

While life expectancy has nearly doubled since Chadwick's report (Woods, 2003, ONS, 2014, Szreter, 1997), social and spatial inequalities in health have persisted. Individuals further down the social ladder still have a disproportionate share of ill health. In our contemporary version of Chadwick's analysis, while we demonstrate that the influence of geographical context has weakened somewhat since Chadwick's era, mortality rates do still vary considerably geographically between occupational groups. Individuals in the ‘intermediate’ occupational group in Rutland had similar or *lower* premature mortality rates than individuals of a ‘higher’ occupational group in the other locations, among both males and females.

The factors influencing health in England in 1842 were very different to those that affected the lives of individuals that died between 2010 and 2012. In 1842 there were problematic environmental inequalities between urban and rural areas, for example, with the adverse environmental impacts of urban areas far more marked in 1842 than today (Szreter and Mooney, 1998, Woods, 2003). Despite these differing contexts, material disadvantage is still a key issue in both periods. Given that we used different measure of mortality, different definitions of social classes, different geographical boundaries and that 175 years have passed, it is remarkable that the rank correlations between the two sets of results remain relatively high. Similar observations have been made over the consistency of Charles Booth's estimates of poverty in Inner London in 1896 and poverty patterns almost 100 years later (Dorling et al., 2000).

What we are detecting in these kinds of analyses is that the same kinds of people tend to live in the same kinds of places over time, and that society still distinguishes people and opportunities by occupation. In effect, our results are evidence that the UK's wider social/economic system hasn't changed much. The persistence in the geography of social disadvantage has been reported widely (Dorling et al., 2000, Dorling et al., 2007, Gregory et al., 2001, Massey and Fischer, 2003). Since this underlying factor is a strong influence on premature mortality rates, it is unsurprising that the mortality patterns have stayed similar.

Our results do not simply confirm these historical trends, but also add to these debates. They raise questions as to *why* individuals of a higher occupational group resident in some areas experience higher mortality rates than among those at lower occupational grades in other locations. Much like the evidence put forward by Chadwick, our study demonstrates the need to consider the interactions between socioeconomic position and geography to understanding health inequalities.

There were multiple strengths and limitations to our study. We utilised administrative data with near perfect coverage for England, which is both novel and useful for estimating population-level patterns. Official mortality records have been under-utilised in previous research, partly due to data accessibility issues, resulting in a reliance on survey data which offer smaller sample sizes. No previous studies have therefore been able to examine whether Chadwick's findings were still relevant. Our analyses are cross-sectional and descriptive which restricts our ability to draw inferences about causality. While we did not tease out the reasons why particular areas may be health promoting or damaging, this was not something we set out to achieve. We were also not able to fully replicate Chadwick's study due to data limitations, and therefore only approximate his measures. One key difference between Chadwick's work and ours was the exclusion of individuals aged below 16. Since infant, child and adolescent mortality were particularly high in 1840s (Woods, 2003), it is plausible that this (and the subsequent narrowing of inequalities in younger ages since 1842) may have contributed to the differences in our results. Chadwick used average age at death in his study, which is a flawed measure of ‘life expectancy’ that could have overestimated the degree of geographical inequalities observed (Hanley, 2002). We also used Local Authorities in 2011 to define our locations. The precise definitions used by Chadwick are unclear and we were not able to define them for a more precise comparison.

In conclusion, our results suggest that geography remains an important factor that influences the extent and magnitude of occupational-based premature mortality rates. It is evident that residence in some locations offers very different life chances and health outcomes for people within the same occupational groups. Chadwick's legacy on the importance of locality is still intact.
